# Spiking neural networks for computer vision

**DOI:** 10.1098/rsfs.2018.0007

**Published:** 2018-06-15

**Authors:** Michael Hopkins, Garibaldi Pineda-García, Petruţ A. Bogdan, Steve B. Furber

**Affiliations:** School of Computer Science, The University of Manchester, Oxford Road, Manchester M13 9PL, UK

**Keywords:** SpiNNaker, spiking neural networks, computer vision, structural plasticity, neuromorphic computing

## Abstract

State-of-the-art computer vision systems use frame-based cameras that sample the visual scene as a series of high-resolution images. These are then processed using convolutional neural networks using neurons with continuous outputs. Biological vision systems use a quite different approach, where the eyes (cameras) sample the visual scene continuously, often with a non-uniform resolution, and generate neural spike events in response to changes in the scene. The resulting spatio-temporal patterns of events are then processed through networks of spiking neurons. Such event-based processing offers advantages in terms of focusing constrained resources on the most salient features of the perceived scene, and those advantages should also accrue to engineered vision systems based upon similar principles. Event-based vision sensors, and event-based processing exemplified by the SpiNNaker (Spiking Neural Network Architecture) machine, can be used to model the biological vision pathway at various levels of detail. Here we use this approach to explore structural synaptic plasticity as a possible mechanism whereby biological vision systems may learn the statistics of their inputs without supervision, pointing the way to engineered vision systems with similar online learning capabilities.

## 1. Introduction

### 1.1. Artificial and biological neural networks

Over the last decade there has been an explosion of interest in the application of deep neural networks [1] and convolutional networks [2] in a wide range of machine learning applications, including computer vision [3]. In parallel, the last decade has seen the development of a number of large-scale neuromorphic computing platforms [4], which support event-based or spiking neural networks. Both of these advances come under the generic heading of neural networks, but the two strands have progressed largely independently from each other. Are there things they can learn from each other?

While all neural networks take a degree of inspiration from biology, those used in today's machine learning have followed a divergent path for some time. They are based upon the multilayer perceptrons of the 1980s, using neurons with continuous-variable outputs, and trained using error back-propagation [5]. The networks themselves are predominantly feed-forward, although some feedback techniques such as long short-term memory (LSTM [6]) are coming into wider use.

Neuromorphic systems have been developed largely to support investigations into the operational principles of biological neural systems and have, therefore, naturally stayed closer to the biology. They use spiking neurons and are trained through local learning rules such as spike timing-dependent plasticity (STDP) [7]. They support strongly recurrent connectivity; in biological systems, feedback connections are as numerous as feed-forward connections.

These differences between the artificial neural networks, that have taken the world of machine learning by storm, and neuromorphic systems, that have stayed much closer to biology, demand further consideration. It is the objective of this paper to explore event-based sensing and processing in the context of computer vision systems and to suggest ways that biological approaches may offer advantages in areas such as rapid and online learning.

The potential advantages of neuromorphic computing are not just of academic and scientific interest; they have also attracted interest and investment from industry, both large and small. The highest profile development has been that of the IBM TrueNorth digital spiking neuron platform [8], joined recently by the Intel Loihi chip [9]. Alongside these established companies there have been several start-up companies, particularly in event-based vision, such as iniVation in Zurich, Switzerland, Chronocam in Paris, France, and MindTrace in Manchester, UK. So there is a growing expectation that event-based systems will play a role in future machine learning applications.

### 1.2. Event-based vision

Nearly all of today's computer vision systems use frame-based vision sensors. These sensors record the entire image that falls upon them many (typically 25–50) times each second. Each image is recorded at uniform resolution. These sensors are highly developed, having evolved from television cameras through various forms of video recording device to the remarkable sensors built into today's mobile phones. The rationale for sensing the visual world in this way is that the transmission or recording is intended to be viewed by a human observer who may be looking closely at any part of the moving image. The frame rate, too, is tuned to the physiology of the human visual system. From the earliest days of movies it has been understood that if a sequence of still images is shown at a suitable rate a human observer will perceive a continuously moving scene.

Biological vision sensors are quite different from frame-based cameras. They do not sample the incident image at a uniform rate, nor at uniform resolution. Different species have differing configurations, but the human eye has a small high-resolution region—the fovea—in the centre of the field of vision, and a much larger vision periphery which has much lower resolution combined with an increased sensitivity to movement. If some unexpected movement is detected in the periphery, the eye is quickly moved to point the fovea at the area of interest for more detailed analysis. In this way, limited resources are deployed to extract the most salient information from the scene without wasting energy capturing the entire scene at the highest resolution. Furthermore, the human eye is primarily sensitive to changes in the luminance falling on its individual sensors. These changes are processed by layers of neurons in the retina through to the retinal ganglion cells that generate action potentials, or ‘spikes’, that propagate through the optic nerve to the brain whenever a significant change is detected. This approach focuses resource on the areas of the image that convey maximum useful information such as edges and other details.

Since the primary goal of computer vision systems is to enable the computer to extract information from the scene, and not simply to record the scene for later human consumption, does it not seem logical to start with a sensor that is more like the biological system than the TV camera?

The frame-based approach to computer vision can, of course, exploit the highly developed state of sensors intended primarily for recording images. The frame-based output contains a huge amount of redundant data and requires ferocious computational power to process, though such power is now readily available. The more biological event-based approach can access event-based sensors, which exist, albeit at a much earlier stage of development. The outputs of event-based devices have lower computational requirements where resources can be focused on salient aspects of the image that are embedded in spatio-temporal patterns of events [10], where again processing algorithms are much less-well developed.

### 1.3. Paper structure

In the remainder of this paper, we continue to make the case for event-based vision. First, we present examples of event-based image processing and motion detection (§2), followed by a brief overview of SpiNNaker, the large-scale machine we have developed for event-based processing (§3). Section 4 describes an algorithm whereby synaptic rewiring can be used to learn the features of visual input, and §5 proposes a speculative approach based on information theoretic principles to model the mechanism whereby individual dendritic branches in cortical pyramidal cells may use rewiring to learn the statistics of their visual inputs. Some suggestions for future work are offered in §6 and our conclusions are discussed in §7.

## 2. Event-based visual processing

Energy efficiency and biological inspiration are characteristics of neuromorphic hardware [11]. Event-based vision sensors (EVSs) take inspiration from vertebrate eyes [12], in particular from the function of photoreceptors which react to changes in illumination [13]. EVSs are cameras whose common feature is to emit events only when they sense sufficient change in the log-luminance [14–16]. These computations are done per-pixel at the transistor level, making them highly efficient; moreover, since events are only generated when a pixel considers the environment changed, transmission energy cost is also low. (This is a simplification of the biological retina, where the outputs from the photoreceptors go through significant processing before they reach the ganglion cells that generate spikes that propagate along the optic nerve.) By having each pixel compute its changes independently, EVSs have a high dynamic range, allowing them to capture everyday scenes better than conventional cameras.

In this section, we consider the processing of event-based visual information. We show how the number of events may be reduced by using a multiscale representation and describe an approach to detect motion in an event-based spatio-temporal stream of visual data.

### 2.1. Image encoding

Although change sensing reduces the number of events the sensor transmits, there may still be too many to send further down the visual pipeline, given bandwidth and energy constraints. Mammalian retinas encode visual information into multiple representations using distinct features [17], likely following the principle of encoding as much information as possible with the fewest signals [18]. There is evidence that some cells in the retina encode luminance information using relative spike times—of the rank-order variant [19]—for which there are models [20,21].

The main mechanism for this encoding is competition between different representations through lateral inhibition. We took this principle, and circuits similar to those in the retina, to create our spiking neural network for image encoding.

**Figure 1.**
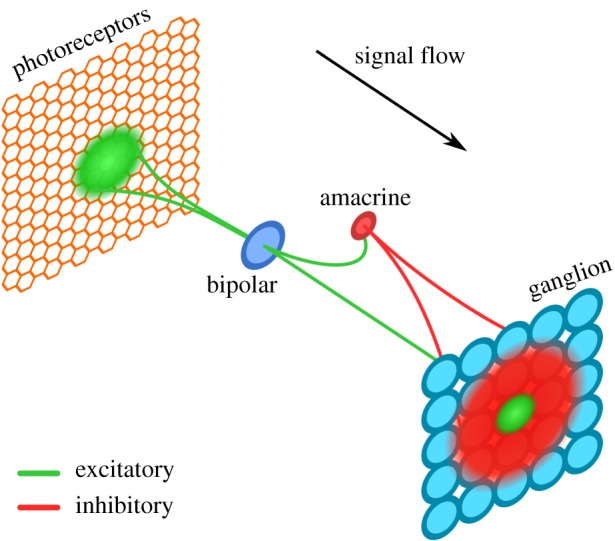
Overview of image-encoding retinal path.

In the first layer of our network (figure 1), bipolar cells sample the input in a nearby region. Synaptic weights are computed according to a two-dimensional Gaussian distribution and stored in a convolution kernel (***W***_*B*_). Incoming weights are then normalized, so that they sum to one, and scaled by the required weight so that a single spike will activate the characterized bipolar neuron; this has the effect of distributing the required activity across the entire receptive field. Each bipolar cell excites a ganglion cell and an amacrine inter-neuron, the latter enforcing competition as it inhibits ganglion cells connected to neighbouring bipolar cells. The weights for inhibitory connections—from amacrine to ganglion cells—are computed by the cross-correlation of the bipolar input kernels,2.1
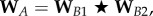
these will also be normalized and scaled as described above. By computing weights in this manner, neighbours will be inhibited proportionally to how similar are the regions they represent. Amacrine cells are inhibitory neurons so their weights (**W**_*A*_) can be considered negative, which means that the total computation (*f*_*G*_) of the circuit can act as a centre-surround filter:2.2
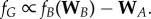

Images are usually composed of elements whose spatial frequency varies, so a single size of input kernel for bipolar cells is not sufficient to encode them efficiently. A bipolar cell whose receptive field has a close fit to an input region should fire sooner than those that fit less well; similarly, its associated ganglion cell will be the first to emit a spike for that region. Additionally, this bipolar cell should suppress activity from neighbouring ganglion cells—at every scale—that might be attempting to represent the input, but with a worse fit.

The competition between different bipolar cells produces a temporal code in which each spike represents a region of the input whose activity matches the receptive field of the spiking neuron quite closely. The receptive fields can be viewed as bases of a vector space; although the bases are not orthogonal, competition will push the representation towards orthogonality. Additionally, the inhibition of neurons representing similar inputs, and the consequent reduction of output spike activity, creates a sparse representation that should provide subsequent stages of the visual pipeline with patterns that are easier to separate.

**Figure 2.**
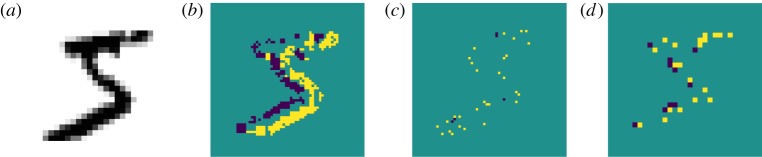
Image processing. (*a*) The input image; this is converted into an event-based representation through an EVS emulator [22] on a host computer. (*b*) An accumulation of events. Events are then processed using the retinal model (on SpiNNaker). (*c*) Accumulated events from the higher resolution filter. (*d*) Accumulated events from the lower resolution filter.

An example of the effects produced by the network is shown in figure 2. Incoming events are accumulated and shown in figure 2*b*, where positive (On) changes are shown in yellow and negative (Off) changes in purple. These events are processed by a version of the image-encoding network, coded in the PyNN neural description language [23,24], with a two-scale configuration. The high-resolution scale uses a 3 × 3 pixel Gaussian kernel with a standard deviation of *σ*_H_  = 0.57 and a spatial sampling frequency of 1. The low-resolution scale uses a 7 × 7 kernel with *σ*_L_ = 0.87 and a stride of 2 pixels. Standard deviation values were selected to cover 3 and 5 pixel diameter regions. Figure 2*c*,*d* shows an accumulation of spikes produced by the high- and low-resolution output units.

### 2.2. Motion sensing

**Figure 3.**
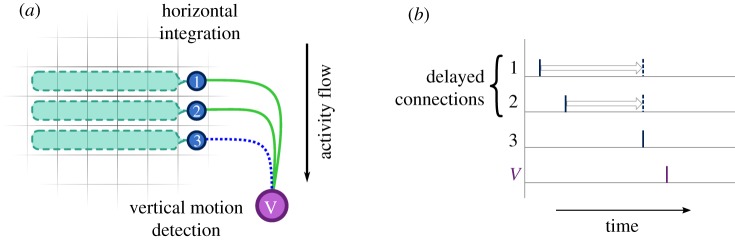
Motion detection architecture. (*a*) Activity from a particular region (cyan) is collected by integrator units (blue circles) and then passed to a detector (purple) through decreasingly delayed lines. (*b*) Spikes are delayed so that they arrive at the same time; this causes a large input to the detector which is then activated. (*a*) Motion detection unit and (*b*) coincidence through delays.

We continue to take inspiration from biological circuits to design a motion-sensing network (figure 3*a*). Bipolar cells integrate regions of the input in an orderly manner (either horizontally or vertically) which then communicate to a motion detector. Motion-detecting neurons usually require two neurotransmitters, one of which has slow dynamics (low-pass or delayed) while the other is present for a brief time. If both signals reach the detector at a similar time, they will induce activation [25].

Delays are inherent to spiking neural networks. If the appropriate delays are applied, the inputs will arrive at the target neuron at the same time [26]. This allows a particular spatio-temporal pattern to be recognized through coincidence detection (figure 3*b*). In our model, slow–fast dynamics are combined with axonal delays to reduce false positives.

**Figure 4.**
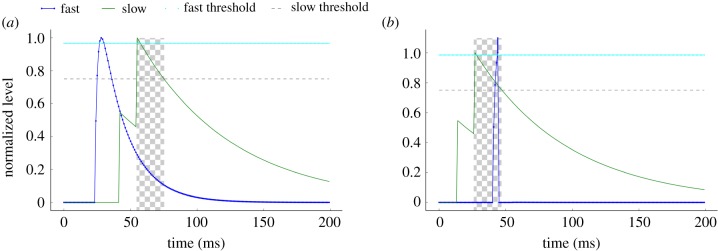
Motion-detection post-synaptic potentials, showing the accumulated slow (500 ms; green) and fast (2 ms; blue-dotted) inputs. If both are above their respective thresholds within a short temporal vicinity (shown by the chequered area), the detector will spike. (*a*) Inverse sequence of events and (*b*) correct sequence of events.

The accumulation of two input events using the slow neurotransmitter (figure 4) opens a 20 ms window (the chequered area) for the fast input to reach its threshold. If the fast-decaying input is received within that window, the detector will fire (figure 4*b*). As only the right sequence of events produces output activity, it is safe to assume that the output indicates apparent motion.

**Figure 5.**
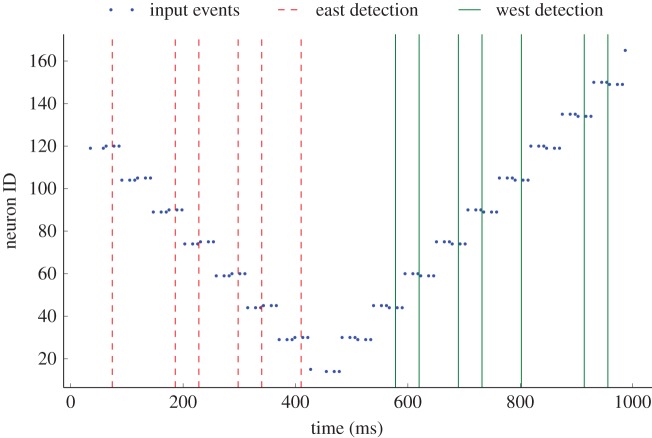
Motion-sensing results. Input events are presented as blue dots; in the first half (until approx. 500 ms), the ball is moving northeast, then the ball bounces off a corner and moves southwest. East motion detection is depicted with red-dashed lines and west detection events are shown as green lines.

To test this, a (5 × 5 pixel) bouncing ball is simulated in a 64 × 64 pixel environment; speed is chosen randomly to be 1 or 2 pixels per dimension per time step. The simulation is rendered into a series of images, which are then converted into events through an EVS emulator [22]. These events are processed by a single-scale centre-surround network using a Gaussian input kernel with *σ* = 0.9, giving a diameter of 5 pixels, and the input is sampled with a frequency of 4 pixels. This processing reduces resolution but provides stability to the input of the motion sensing units by reducing the number of events per region. Figure 5 shows the results of the simulation in terms of motion detection. The left half of the figure corresponds to the ball moving in a northeasterly direction, and the corresponding easterly motion detection events (red-dashed vertical lines). The right half of the figure shows the ball moving southwest with westerly detection activity (green vertical lines).

## 3. Event-based processing: SpiNNaker

The event-based visual processing networks described above are suitable for running (and, indeed, have been run) on a neuromorphic platform. SpiNNaker (Spiking Neural Network Architecture [27]) is one example of a large-scale neuromorphic system. It is based upon a bespoke many-core chip; neuron and synapse models are implemented in software, in contrast to most other neuromorphic systems where those models are implemented using analogue or digital hard-wired circuits. The software approach has strengths and weaknesses: its major strength is flexibility, as new models and learning rules may readily be added to the software libraries; the downside of this approach is that software inevitably incurs an energy-efficiency overhead of around one order of magnitude. This makes SpiNNaker well suited to use as a research and development platform, whereas hardware algorithms are more efficient as the basis of an application delivery platform.

The neuromorphic aspect of SpiNNaker is the way the processors are connected. Biological neural networks display very high degrees of connectivity, with neurons often having many thousands of inputs, sometimes as many as quarter of a million. Here SpiNNaker borrows the well-established neuromorphic technique of address event representation (AER [28,29]), wherein each neuron is given a unique numerical ‘address’, but instead of employing AER on a broadcast fabric it is mapped onto a packet-switched fabric, thereby improving system scalability.

**Figure 6.**
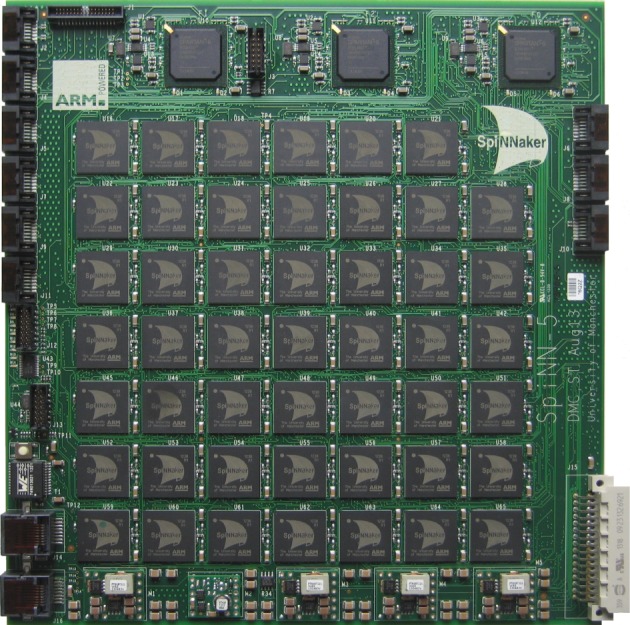
The 48-node, 864-core SpiNNaker circuit board. (Online version in colour.)

**Figure 7.**
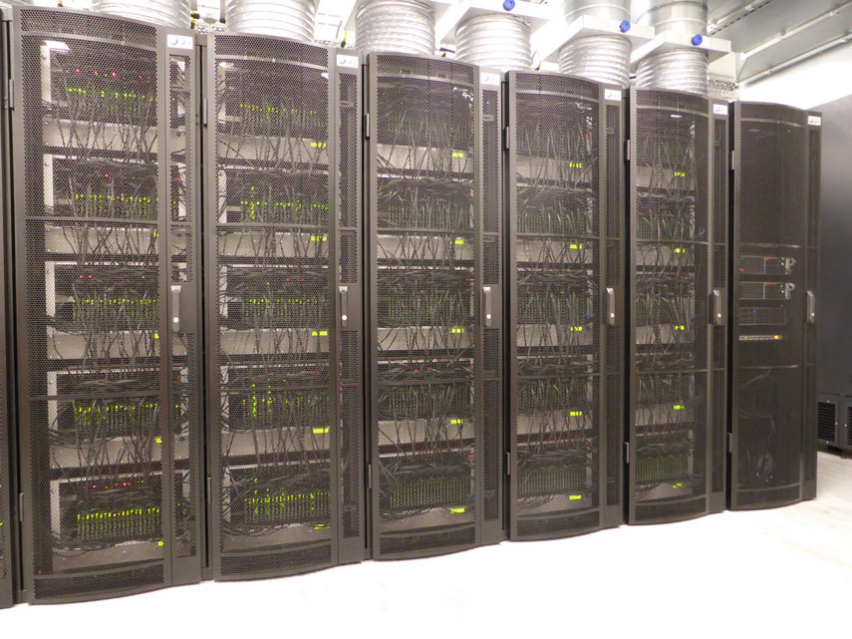
The 500 000-core SpiNNaker Human Brain Project platform. (Online version in colour.)

SpiNNaker hardware has been delivered at a number of scales, from the small 4-node (72-core) board that can model networks of a scale equivalent to a pond snail brain, through the 48-node (864-core; figure 6) board that can model networks of small insect scale, up to the 500 000-core machine that forms the basis of the SpiNNaker platform offered openly under the auspices of the European Union Human Brain Project (figure 7). Each processor core can be used to model a few hundred spiking neurons and around a million synapses forming the inputs to those neurons.

**Figure 8.**
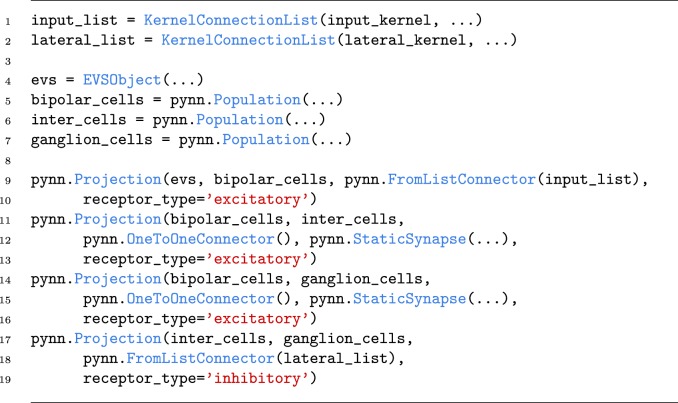
Multi-scale image representation PyNN code. (Online version in colour.)

SpiNNaker has support software [24] which maps a spiking neural network written in PyNN [23] onto the machine. A simplified PyNN description of the multiscale image processing network described in §2.1 is shown in figure 8 to illustrate the style of the description. The complete PyNN model can be found in the data repository associated with this paper.

## 4. Structural plasticity for classification

With an event-based vision stream, pre-processing based upon a retinal model, and a suitable neuromorphic platform such as SpiNNaker, we can then proceed to perform additional processing to achieve outcomes such as object recognition and classification. In the following sections, we discuss some of the principles of visual processing in the brain, and some experimental work to develop object classification systems based upon those principles.

### 4.1. Topographic maps

A widely observed principle in biological brains is the use of topographic maps, wherein two-dimensional topological (though not necessarily scale) relationships are preserved in projections from one brain region to another.

**Figure 9.**
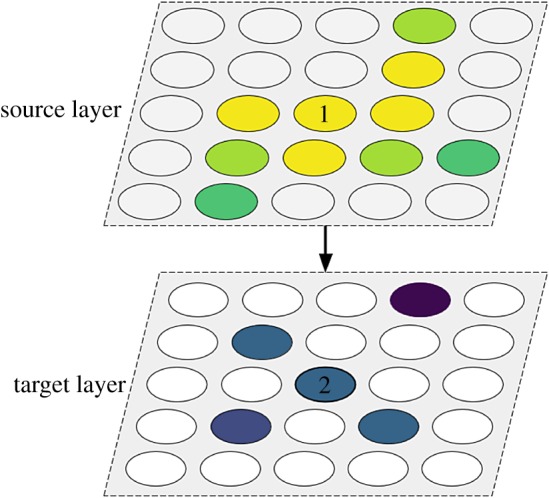
Topographic maps. Neuron (2) in the target layer has a receptive field formed by connections from the source layer (feed-forward) as well as connections from within the target layer (lateral). These connections are centred around the spatially closest neuron, i.e. neuron (1) in the case of feed-forward connections. Connections from more distant neurons are likely to be weaker (indicated by a darker colour). (Online version in colour.)

Neural topographic maps consist of layers of neurons whose reaction to afferent stimuli changes with area (figure 9). Such an organization is characterized by the preservation of neighbour activity from the source to the target layer and provides several advantages in terms of wiring and information processing and integration. Wiring is optimized since neurons generally have limited receptive fields and tend to be interested in spatially clustered locations. As an example, orientation-selective neurons, such as those present in primary visual cortex, are required to have afferents from small regions of the total visual receptive field, thus a topographic organization ensures that neurons only connect to their immediate neighbours and have limited interaction with those which are further away. More importantly, when neurons form multiple aligned maps, each receiving information from a different modality, they exhibit multisensory facilitation; their response is supra-linear if they receive synchronous stimuli from the same area of space arriving from different modalities. This is the case in the superior colliculus, a brain structure which integrates signals from multiple senses and also guides adaptive motor responses [30].

Topographic projections are widespread in the mammalian cortex [31]. Their development has been explored through simulation, with and without spiking neurons, and involving both synaptic plasticity [32–34] and synaptic rewiring [35]. The latter example has been modelled on SpiNNaker. It is with that model we suggest an architecture capable of handwritten digit classification through supervised learning.

In short, the suggested model involves the cooperation of two types of mechanisms: activity-independent and activity-dependent. The former is represented by the formation rule: it relies on the distance between potential partnering neurons in order to create a new synapse—neurons which are spatially clustered will tend to form more connections than neurons which are spatially distant. The latter is composed of two mechanisms: STDP, and a removal rule for the synaptic rewiring mechanism. STDP, using local spiking information, modifies the weights of synapses connecting neurons together, while the elimination rule preferentially removes those synapses which are depressed—synapses which carry ‘useful’ patterns or subsets of patterns to neurons will tend to be reinforced, thus are more stable in the long term, conversely, synapses which usually transmit what amounts to noise will be silenced and more likely to be pruned. All of the aforementioned mechanisms operate continuously, at a fixed rate, on a population of neurons.

The model we use differs from the one suggested by Bamford *et al.* [35] in several salient respects:

— it is simulated in real-time on the SpiNNaker neuromorphic hardware;
— more realistic input (MNIST digits) at larger spatial scales is provided to each target layer;
— connections are generally static (weights are not modified by STDP);
— lateral connections (target-target) are inhibitory, rather than excitatory;
— simulation of mechanisms for different purposes: Bamford *et al.* proposed the model as a mechanism for topographic map refinement; here we suggest a different use: digit classification.

### 4.2. Network architecture

In the context of spiking neural networks, learning is usually associated with the longer term increase or decrease in the efficacy of synapses. Such an increase could occur because a synapse has detected a pre-synaptic action potential followed closely by a post-synaptic one. Conversely, a post-synaptic action potential followed by a pre-synaptic one is considered anti-causal and the synapse processing the events decreases its efficacy. STDP is the mechanism most usually modelled to induce long-term potentiation or depression [7,36].

However, a network can learn even without a change in the efficacy or weight of a synapse. Using structural plasticity on SpiNNaker, a network can solve the task of classifying handwritten digits either using only static synapses, or STDP can be used to modulate the synaptic rewiring. The synaptic rewiring model includes two probabilistic rewiring rules [35]: one for synaptic formation, the other for elimination. Formation is a probabilistic, activity-independent process dependent on the distance between candidate neurons. A new synapse is formed with maximum weight 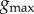 if4.1
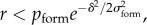
where *r* is a random number sampled from a uniform distribution in the interval [0, 1), *p*_form_ is the peak formation probability, *δ* is the distance between the two cells and *σ*^2^_form_ is the variance of the receptive field. The result is a Gaussian distribution of formed synapses around the ideal target site, i.e. around the target neuron where *δ* = 0.

Removal is either carried out with a fixed probability *p*_elim-pot_ when weights are static, or with a choice of *p*_elim-pot_ or *p*_elim-dep_ when applied in conjunction with STDP. Thus, a synapse is removed if4.2
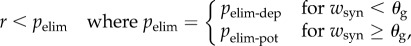
where *r* is a random number sampled from a uniform distribution in the interval [0, 1), *p*_elim-dep_ is the elimination probability used when a synapse is depressed, *p*_elim-pot_ is the elimination probability used when a synapse is potentiated, *w*_syn_ is the weight of the synapse under consideration for removal and a weight threshold *θ*_*g*_ is selected as half of the maximum allowed weight (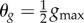). If STDP is not presented in the simulated network, only *p*_elim-pot_ is used as all synapses would have a fixed weight, namely 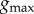.

**Figure 10.**
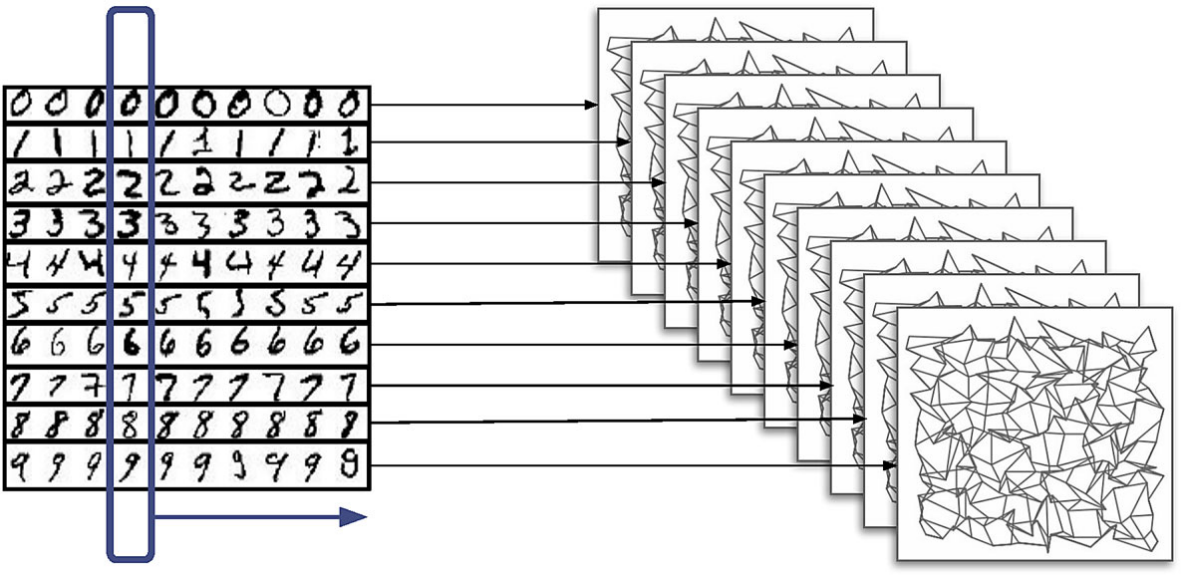
Network architecture used for training. A source layer displays a series of examples of handwritten digits; each example from a particular class is projected to the target layer corresponding to that class. (Online version in colour.)

The model is equivalent to the supervised learning paradigm in artificial neural networks. Data are labelled using a dedicated projection from a source layer to the corresponding target layer. A layer of neurons providing examples belonging to a class connects exclusively to a population which learns to recognize members of that class. Figure 10 shows the network architecture of the training regime, where each source in a source-target population pair displays a digit for 200 ms for a total of 300 s; the initial connectivity between each source–target pair is 1%.

**Figure 11.**
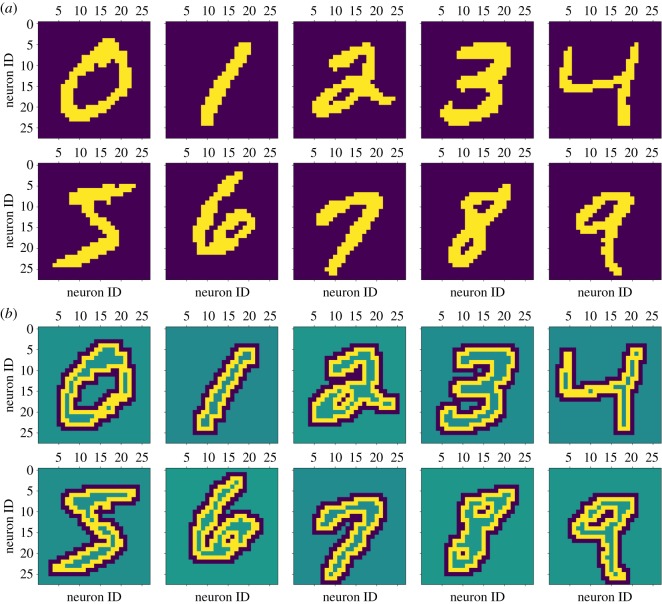
Example inputs presented from each source layer to the corresponding target layer before transformation into a rate-based representation and adding noise. (*a*) Digit shape before the application of the centre-surround filter. (*b*) Edge information transmitted as ‘On’ pixels (a positive change in brightness, lighter colour) and ‘Off’ pixels (a negative change in brightness, darker colour). The network has been tested with both types of input. (Online version in colour.)

To generate the input, each original digit is filtered via convolution using a 3 × 3 centre-surround kernel, mimicking the response of the highest resolution retinal ganglion cells. The kernel is normalized to sum to zero with an auto-correlation equal to one. Finally, a threshold is applied after the convolution operation, resulting in edge-detection. Transmission within the network is achieved through the use of neurons which generate Poisson spike trains; each pixel within the 28 × 28 image is mapped to two Poisson neurons, one for the on and one for the off channel. Figure 11 shows examples of input digits before adding background noise; all of the feed-forward connections are excitatory.

Neurons within each target layer also receive lateral connections. These are inhibitory and their main purpose in this network is to limit the spiking activity within the target layer. While connections within each target layer abound, there are none between the layers.

The network is described using the PyNN [23] simulator-independent network description language. The SpiNNaker, simulator-dependent, implementation of PyNN (sPyNNaker [24]) has been extended to perform synaptic rewiring. Table 1 (mirroring, with only slight modifications, table 1 from [35]) contains the parameters used in the simulations presented in this section. The wiring parameters affect the synaptic rewiring mechanism and its operation, from the rate at which rewiring occurs (*f*_rew_) and the size of each neural layer (*N*_layer_), to the individual formation and elimination probabilities used (*p*_form_ and *p*_elim_) and the maximum number of possible afferents that an individual neuron can have (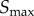); we distinguish between feed-forward (ff) and lateral (lat) connections. The Poisson neurons which, in conjunction, transmit the digits to the target layers (after a delay of 1 ms), fire with an overall mean firing rate of *f*_mean_ = 5 Hz, and display each digit for *t*_stim_ = 200 ms. Finally, the behaviour of individual leaky integrate-and-fire neurons and conductance-based synapses is controlled by the membrane and STDP parameters, respectively. The behaviour and choice of these parameters is further explained by Bamford *et al.* [35].

**Table 1.** The parameters used in the simulations presented throughout this section.

wiring	inputs	membrane	STDP^a^
*N*_layer_ = 28 × 28	*f*_mean_ = 5 Hz	*v*_rest_ =−70 mV	*A*_+_ = 0.1
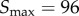	*t*_stim_ = 200 ms	*e*_ext_ = 0 mV	*B* = 1.2
*σ*_form-*ff*_ = 2.5	—	*v*_thr_ =−54 mV	*τ*_+_ = 20 ms
*σ*_form-*lat*_ = 2	—	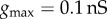	*τ*_−_ = 20 ms
*p*_form-*ff*_ = 0.16	—	*τ*_*m*_ = 20 ms	—
*p*_form-*lat*_ = 1	—	*τ*_ex_ = 5 ms	—
*p*_elim-dep_ = 0.0245	—	—	—
*p*_elim-pot_ = 1.36 × 10^−4^	—	—	—
*f*_rew_ = 10 kHz	—	—	—

^a^The STDP parameters are only used when synaptic plasticity is used in conjunction with the rewiring.

### 4.3. Classification

**Figure 12.**
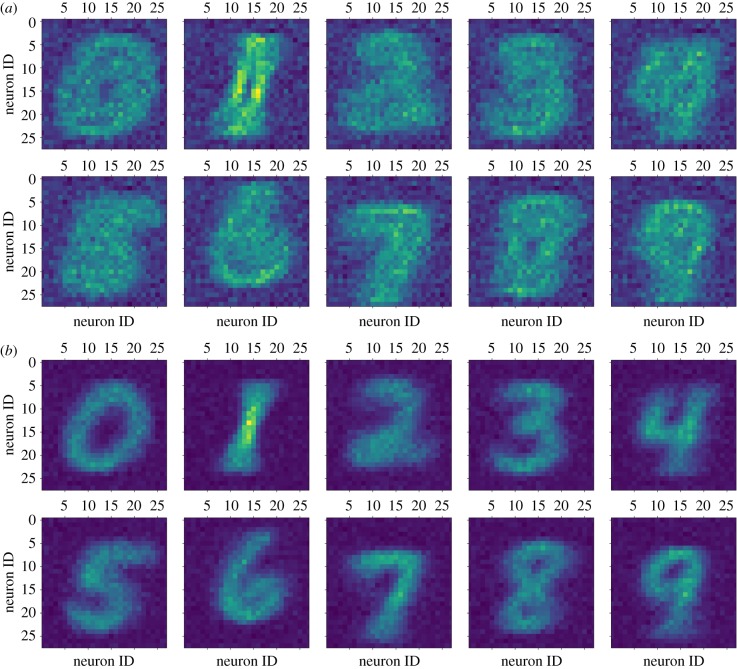
After training, an average representation of each input digit class can be reconstructed from the individual source population fan-out patterns when presented with filtered (*a*) and non-filtered (*b*) inputs. In the filtered input case, the image is built from summing together the reconstructed connectivity from the ‘On’ and ‘Off’ source layers. Brighter colours equate to more connections originating from that pixel. The current plot shows only the effect of synaptic rewiring; weights are not included. (Online version in colour.)

Figure 12 shows that it is possible to identify visually what each target layer has learnt; time-averaged digits from each class are embedded into the connectivity of the network. It is then possible to test the quality of classification. For this, we make use of a single source layer, or a single pair of source layers in the case of the filtered digits: one represents ‘On’ events, the other represents the ‘Off’ events. The previously learnt connectivity is used to connect all of the target layers to the source layer, and all plasticity is disabled. The source layer now displays class-randomized examples, each for 200 ms. The classification decision is made off-line, based on which target layer has the highest average firing rate within the 200 ms period.

This is not a state-of-the-art MNIST classification network (it achieves a modest accuracy of 78% and an RMSE of 2.01 with non-filtered inputs, performance drops when filtered inputs are used: an accuracy of 71% and an RMSE of 2.38) as each input digit class is represented only as an average for that class, but it serves here to demonstrate that synaptic rewiring can enable a network to learn, unsupervised, the statistics of its inputs. Moreover, with the current network and input configuration, the quality of the classification is critically dependent on the sampling mechanism employed in the formation of new synapses. Random rewiring, as opposed to preferentially forming connections to neurons that have spiked recently, could achieve accurate classification only if operating in conjunction with STDP or some other mechanism to prevent the pruning of useful synapses; such a mechanism is discussed in §5.

## 5. Dendritic branches and information theory

An alternative view of the same problem is to consider how the visual system (and cortex more generally) could adapt most efficiently to the inputs that it receives so that it can use limited resources to span and represent the input space of most interest. One can see this as a classical unsupervised learning problem where, in this context, it would mean building a representation of the input distribution, or perhaps a prior distribution for a Bayesian inference mechanism.

In the last 15 years, it has become increasingly clear that the point neuron model which has dominated artificial spiking neural networks does not paint the whole picture of how neurons learn and respond to the inputs on their dendritic tree [37–41]. The term for this area of study is *dendritic computation* and there is ample evidence from these references, in particular, which suggests, among other things, that

— synapses tend to cluster on dendritic branches;
— these clusters can operate as independent processing elements;
— they demonstrate potentially useful properties such as adaptation, nonlinear response to sparsely coded synaptic input, homeostasis and the generation of NMDA potentials (electrogenesis mediated by NMDA receptors) which travel along the dendritic branch.

If this is the case—which now seems likely—it means that the input to the soma is heavily pre-processed, with the implication that any firing of the neuron is in response to a far more nuanced spatio-temporal pattern at the synapses than is possible with a simple linear sum, whether or not a sigmoidal or other nonlinearity is applied. So the implication is that a neuron is a more sophisticated processor than previously thought, with each action potential produced containing a higher ‘information content’.

Stepping back from the detail, a more general view of the cortical mechanism under consideration here is that of a hierarchical inference system which uses sparse codes for learning and applying spatio-temporal patterns robustly in the presence of noise and component failure or variation. The lower layers of the hierarchy deal with features that are faster-moving and more detailed and, as one goes higher in the hierarchy, they become temporally more stable and ‘spatially’ more abstract. Arguably, the great recent success of *Deep Learning* as described by LeCun *et al.* [42] is at least partly due to this hierarchical conception.

A further speculation is that individual neurons use a combination of *unsupervised learning* for building prior models of likely input in the dendritic branches—perhaps especially so in the apical tuft where a backpropagating action potential (bAP) will rarely be present in the human pyramidal neuron—and *supervised learning* in or near the soma where a bAP signal is available to incorporate targets, training signals and rewards, which are either immediate, or delayed in the case of *reinforcement learning*.

### 5.1. Assumptions and a simple test

A clear opportunity from the above perspective is to understand how each dendritic branch can recognize and adapt to input, and then potentially send on information, in the form of an NMDA potential, when certain spatio-temporal patterns are recognized. Simplifying the problem further in order to provide analytical tractability, one can see the inputs as binary variables where an action potential from the retinal subsystem (itself significantly pre-processed as described in earlier sections) is either present or absent in any given time step. A natural choice of time step could be the refractory period of a neuron, i.e. ≈ 3 ms which would ensure binary output codes, but this is by no means a requirement. This set of simplifying assumptions means that one can then view the information transfer mechanism in cortex as binary codes, and there is both evidence from neuroscience and tangible computational benefit if these codes are *sparse* [18,43–48].

We recognize that the approximations and mechanisms described in this section are partly speculative but they are based upon a consideration of results and patterns seen in the references provided. We do not claim the mechanisms to be exact, although it is interesting to note that the Maximum Entropy sampling algorithm described below is replicated exactly in a recent paper providing arguably the most rigorous and comprehensive application of information theory to brain function (Parr & Friston [49], 3rd element of the full Generalized Free Energy equation in appendix A).

To assess the unsupervised mechanisms suggested above, the original MNIST binary dataset has been used as a test problem to see how simple but biologically plausible homeostasis and learning mechanisms in dendritic branches would adapt to their input distributions. Work is ongoing to extend these ideas using similar information theoretic principles to judge the classification performance of a supervised learning mechanism which uses the output from these dendritic branches. As far as possible, it is desirable that this mechanism should be feed-forward and online, i.e. without the need to resort to neurally implausible mechanisms such as error backpropagation using chain rule derivatives and many repeated epochs of batch learning.

The input to the sampling subsystem is 60 000 binary input patterns across the 28 × 28 input raster plot with each digit accounting for approximately 6000 examples. The global input density of this dataset is shown in figure 19*a*.

The processing elements are individual dendritic branches containing a cluster of related synapses. Mel [37] give evidence that clusters of five to nine synapses have certain desirable properties and they use eight in their more detailed study. With our aim (described later) of achieving a firing probability *p* ≈ 0.5 for each branch we have chosen *n* ≈ 20 somewhat arbitrarily—it may be that a smaller number is more effective. Let us consider one dendritic branch, which will be defined as a section of dendrite structure with some number of ‘local’ synaptic connections. It has been shown that both memory and computation are potentially available within a branch, as well as the possibility of communication between local branches. Such locality is of great benefit—perhaps even essential—for realistically large networks of synapses and neurons to be able to learn. We are going to further assume binary behaviour, i.e. at any given point in time a synapse is either 1 (firing) or 0 (silent). If a branch has 20 synapses, it can therefore provide a binary code with 2^20^ possible states. This can be seen as a sparse code as it samples a much larger input space (in MNIST 784 pixels) leading to a 20-of-784 code as described by Furber *et al.* [44].

The distribution of the behaviour of synapses in the branch will be *multivariate Bernoulli*. As shown by Dai *et al.* [50], this distribution is a member of the exponential family of distributions and hence a *Maximum Entropy* distribution [51,52] with attendant advantages in terms of both analytical simplicity and when one wishes to reason about the information-theoretic behaviour of the branch. Some interesting properties of this distribution are

**Figure 13.**
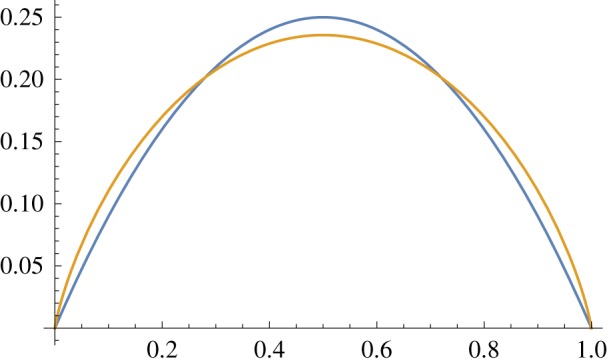
(Scaled) entropy in gold and variance in blue; as a function of probability. (Online version in colour.)

(1) *independence* and *uncorrelatedness* are equivalent—which is not generally true of multivariate distributions;
(2) *variance* and *entropy* of the distribution are either exactly or approximately equivalent in the sense of being similar convex functions of *p* from 0 to 1 (figure 13);
(3) both marginal and conditional distributions of subsets of the distribution are still multivariate Bernoulli.

Points 1 and 2 above make calculations with variances (and covariances) essentially equivalent to calculations about entropy, but much easier to carry out.

Each branch should sample the input receptive field ‘most effectively’ in some well-defined sense. Some desirable properties could be

— focusing on the input distribution so that more resources are allocated where input is more likely—this can be seen as *importance sampling*, *density estimation* or *unsupervised learning* depending upon your point of view;
— extracting the most ‘signal’ in the presence of inevitable ‘noise’, i.e. maximizing the signal-to-noise ratio;
— capturing ‘features’ in the input signal.

An approach that combines these desirable features is described in the experimental design community as *Optimal Design* [53] where a mathematically tractable criterion is maximized by the choice of input samples.

The original thinking about such optimal sampling mechanisms was in the context of Variance- or Alphabetic-optimal experimental design for regression modelling (e.g. [53,54] and for an insightful practical analysis ch. 14 of [55]). A nice generalization of the ideas to other models called *Maximum Entropy Sampling* incorporates information-theoretic principles more explicitly [56].

Much work has been done on algorithms for finding the best sampling design in these problems [57]. Usually, these are formulated as *exchange algorithms* where a candidate point in the current sample is dropped and a different candidate point is added to see if the chosen criterion is improved. This continues until convergence. In some cases, the problem is convex and converges quickly and robustly, in others a global optimization method such as simulated annealing is required in order to avoid the many local optima. In almost all cases, matrix computations are required with the criterion usually being some function (e.g. the determinant) of a covariance matrix of the posterior parameter distribution in the case of a linear or nonlinear regression model, or perhaps of the posterior conditional Gaussian Process.

As indicated in point 2 above and the comment afterwards, for a multivariate Bernoulli distribution the entropy of the distribution of joint probabilities is equivalent to the *generalized variance* which—as in the multivariate Gaussian case—is a scalar monotonic function of the determinant of the covariance matrix (|Cov|), reducing to a simple variance in the univariate case. So a first idea to investigate is to find out what would happen if we choose synaptic connections so that our dendritic branch samples from the input in order to maximize the source entropy. This would be equivalent to maximizing the determinant of the covariance matrix of samples from the training set. The best possible outcome is for all the variances on the diagonal to be their maximum value (0.25 for a Bernoulli variable implying a probability of 0.5) and all off-diagonal entries to be zero indicating no correlations between any of the sampled points. This is unlikely to be feasible in practice but for reference with *n* = 20 the value of ln|Cov| in this case is −27.73. The most direct intuition for this is the measurement of a hypervolume in 20D space. One can compare this to the values attained in figures 15 and 17. For example, the maximum value found in figure 17 is ≈−33. Comparing the two hypervolumes gives a ratio of 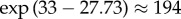 times smaller which sounds huge until considering that in 20D space one would only need to reduce each axis by 
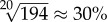
 to make this reduction in hypervolume.

In terms of computational neuroscience this is *structural plasticity*, i.e. we are altering synaptic connectivity in order to improve the sampling properties of the dendritic branch. In this context, the covariance matrix of input samples over the training set will capture binary, ternary and higher-order relationships between pixels that relate to features that are present and of direct interest in the training data. It should be noted that, for the moment, all input connections to one branch must represent different pixels.

### 5.2. Some results

A first look at the problem required computation of the full 784-dimensional covariance matrix of the 60 000 MNIST training inputs. The original unaliased formulation is used, i.e. a pixel is either present or absent. In the dataset available, where 8-bit greyscales have been generated, a cut-off point of 80 has been chosen, above which a pixel is **on** and below which it is **off**.

**Figure 14.**
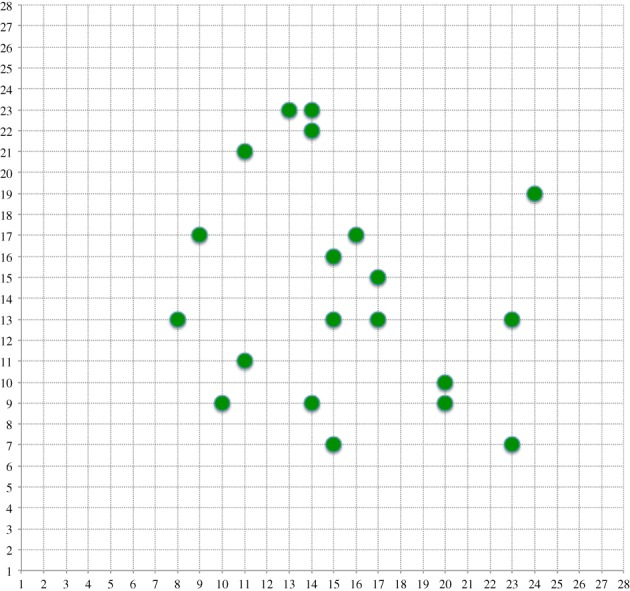
Position of sampled inputs after 1M random selections using |Cov|. (Online version in colour.)

Now a simple algorithm was used to choose a 20 × 20 covariance submatrix by extracting the appropriate rows and columns from the full matrix, in order to maximize its determinant. A simple random search retaining ‘best found so far’ was used and as expected a slow progression towards better values was seen. Figure 14 shows the best point set selected after 1M random choices. Clearly, they are in the higher density region and show some clustering.

A second approach is like a simple exchange algorithm where the pixel *k* to be removed is the one with the smallest value of a summation of absolute values of the covariance matrix of the current input connectivity thus5.1
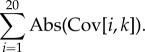


The reasoning behind this choice is simplicity; more sophisticated ways of choosing a point for removal are available in the literature and these are likely to have better ultimate performance, but this is not our goal here. A side-effect of this choice mechanism is that a sample with lower values off the diagonal (i.e. lower covariances) will tend to be chosen for removal, which is not ideal.

**Figure 15.**
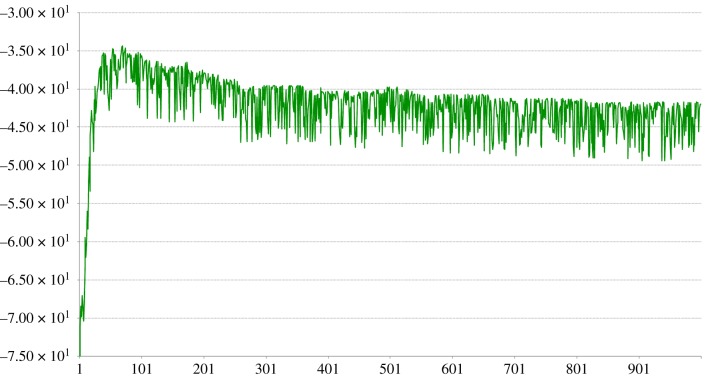
Change in value of ln|Cov| over 1000 replacements using the method in equation (5.1). (Online version in colour.)

Replacing the rejected connection this way with another randomly chosen pixel provides an apparently effective approach, with figure 15 showing a fast rise to a close-to-optimal value for the determinant after something like 30–70 exchanges, followed by a slow and gentle fall to a stable value that is still significantly higher than the random search mechanism was able to find after 1M random draws.

It did not seem to matter whether one started with a good randomly chosen starting set or not, which bodes well for the robustness of such an algorithm.

**Figure 16.**
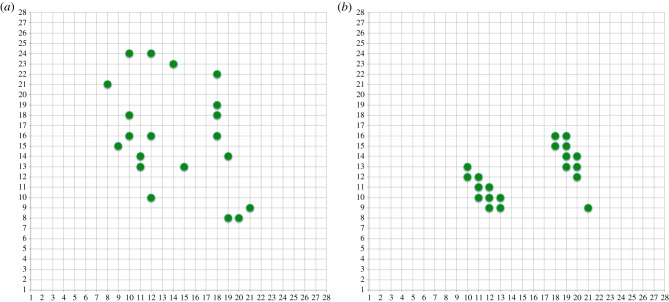
Position of sampled inputs after (*a*) 69 and (*b*) 1000 replacements using |Cov|. (Online version in colour.)

Figure 16 shows the final pixel sampling positions after 69 exchanges (the one leading to the highest |Cov| value) and after 1000 exchanges when the algorithm has presumably ‘bedded in’ and done some wandering around a solution space where the |Cov| value is sacrificed to some extent by competing aspects of the algorithm—this is discussed below.

Figure 16*a* looks similar to the randomly generated solution above but with a significantly better |Cov| value and probably with more clustering; figure 16*b* is clearly very clustered and in fact generates something that looks very much like two randomly oriented Gaussian patches. This is food for thought considering the amount of work on such receptive fields in the visual input system [58], because here they have arisen entirely from a Maximum Entropy sampling exchange algorithm applied to realistic input.

### 5.3. A more biologically plausible algorithm

As far as we know, the brain is not designed to carry out the type of computation required for replicating the above result and even if it has evolved mechanisms for calculating simple linear algebra operations such as vector–matrix multiplication, it is highly unlikely that complex and numerically sensitive operations such as matrix determinants and inverses are feasible with the computing machinery available. So it would be useful to find a simpler and more biologically plausible mechanism that could be used to produce a similar result. It seems that such mechanisms do exist if we allow some very simple computation and storage to be carried out within the dendritic branch. The following mechanism was chosen that we believe is simple enough to be biologically plausible. Each synapse is given an integer value; this does not relate to its effect on the branch computation but defines its longevity, and when it gets too small it means the synapse has atrophied and will be replaced by another randomly chosen connection. This makes sense in terms of how structural plasticity has been observed, and something similar has been suggested by Numenta in their HTM implementation [46].

At any time step then, some number of the synapses in the branch will receive input from their receptive field and fire. If this number is above an adaptive homeostatic threshold, an NMDA potential is generated and the synaptic longevity is increased by 1 if the synapse was involved in the latest spike generation or reduced by 1 if not. A lower limit is established in advance so that the synapse with smallest longevity (i.e. the most atrophied one) is only replaced if the longevity falls below this limit. This is the only ad hoc tuning parameter in the mechanism so far and adjusts the balance between stability and the number of synapses recycled. This parameter could itself be stochastic and/or adaptive to aim for a target stability. Fortunately, the mechanism does not appear to be sensitive to any of these choices which bodes well for robust performance.

This simple mechanism has a number of sensible properties:

— from neuroscience—it resembles Hebbian plasticity in that those synapses which fire together are strengthened;
— from information theory—it tends to promote a maximum generalized variance by choosing samples with individually high information content (though see below for a caveat);
— one can make arguments for biological plausibility from the dendritic computation literature via local processes within the branch;
— it is to a certain extent self-stabilizing as there will always be synapses that do and do not fire from changing input.

**Figure 17.**
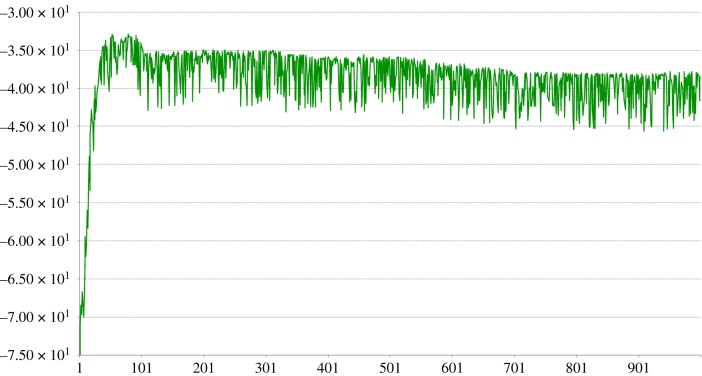
Change in value of ln|Cov| over 1000 simple dendritic branch computation replacements. (Online version in colour.)

**Figure 18.**
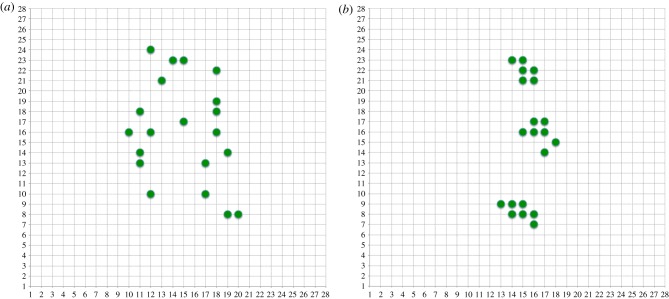
Position of sampled inputs after (*a*) 69 and (*b*) 1000 replacements using simple dendritic branch computation. (Online version in colour.)

For ease of programming, the results in figures 17 and 18 are generated using the entire training set for making a change in synapse quality but it is simple enough that an online incremental version will be easy to implement. They are, therefore, equivalent to the results above from the direct matrix computations.

Comparing the plots generated from this very simple algorithm to the earlier ones, a few things can be noted:

— the |Cov| values are uniformly better;
— the trend over replacements is very similar;
— the structure of the sampling points after the early finish at maximum |Cov| and after 1000 replacements are both very similar.

So this version of the algorithm seems to perform at least as well as the matrix version, arguably better.

As mentioned above, one caveat needs to be noted. A true Maximum Entropy sampling algorithm (or D-optimal experimental design which is conceptually equivalent) would maximize the diagonal variances and minimize the off-diagonal covariances in order to maximize the generalized variance—which in the multivariate Gaussian case is equivalent to a hypervolume defined by the covariance matrix and measured by its determinant. The closest that we get to this in the above experiments are the samples where the |Cov| value is highest, i.e. figures 14, 16*a*, 18*a*. In these cases, the off-diagonal terms will tend to be smaller promoting independence between the individual synaptic samples. However, due to how we choose samples for removal in the first algorithm using equation (5.1) and how the synaptic replacement mechanism is formulated in the second, the samples move away from this local maximum. This is because both of these mechanisms will tend not to penalize samples that are correlated, i.e. they will allow the off-diagonal terms in the covariance matrix to increase. This may well be useful in terms of finding features in the input sample (in itself a valuable outcome), but it moves away from a pure Maximum Entropy sampling method. Two intriguing questions are then:

— would a small change to the algorithms stay closer to a true Maximum Entropy sampling method?
— can one identify and exploit the trade-off between maximizing generalized variance and finding features?

**Figure 19.**
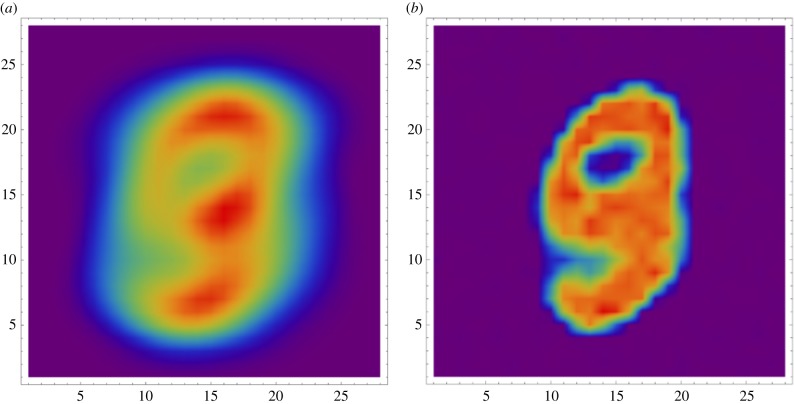
(*a*) Input density from 60 k MNIST binary training data. (*b*) Sampled input density using the simplified online algorithm and 2500 branches. (Online version in colour.)

Figure 19*b* shows the combined density of 2500 branches after training. Clearly, the input density is related to the known input density in figure 19*a*, focusing on populated input pixels and ignoring unpopulated—one important feature of unsupervised learning. It does not sample the less populated areas as much as the real input density.

### 5.4. Properties of the branch outputs

In the dendritic computation literature, a widespread view is that when a large enough number of the local synapses fire, an NMDA plateau potential is generated. Ignoring for a moment the timing issues (these plateau potentials are much longer than somatic action potentials) we can then choose how and when such a dendritic branch output should be generated. Following the information-theoretic rule that the maximum information available from a binary variable is when *p* = 0.5 (see, e.g., [47] and figure 13), and also bearing in mind the very important issue of homeostasis which is likely to be a crucial property for any neural processing system, a simple adaptive thresholding mechanism has been built into the branch to ensure that *p* ≈ 0.5 indefinitely. Of course, this is not necessarily neurally plausible and some trade-off in biological systems is almost certainly required in order to reduce energy consumption by biasing the firing probability towards 0 from this optimal value. There are also arguments about sparsity of coding that would suggest a firing probability lower than 0.5. This is a potentially important issue that requires further investigation.

**Figure 20.**
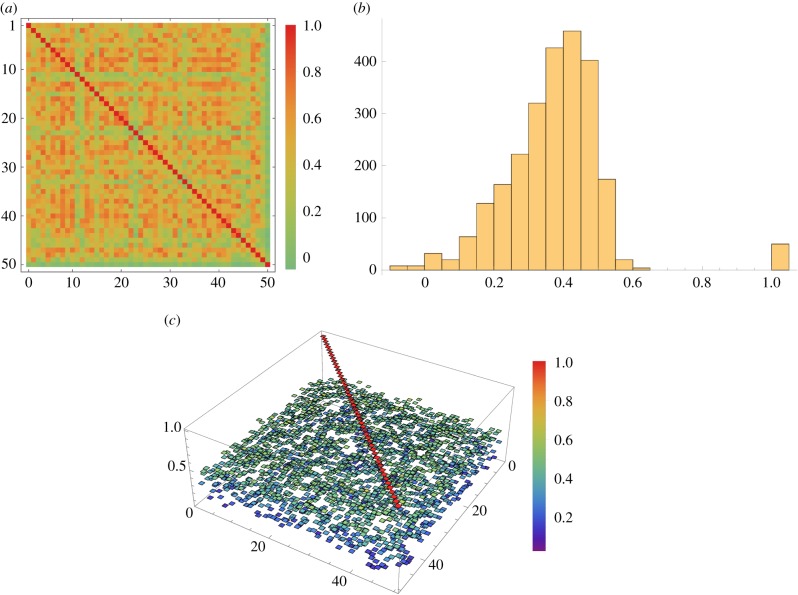
Some views of the correlations between outputs from 50 independently generated branches. (*a*) Two-dimensional view of correlations, (*b*) histogram of correlations and (*c*) three-dimensional view of correlations. (Online version in colour.)

It will also be important to confirm that each branch does not converge onto the same input sample, as multiple branches are only of use if they incorporate different information. This may require some thought, but the literature does suggest that communication between local branches is possible and hence some simple inhibitory mechanism could be fashioned if necessary. To test the simple and currently independent set-up we look at the correlations between NMDA potentials from 50 independently generated branches after training is complete. A perfect choice of orthogonal features would of course have 1 on the diagonal and 0 elsewhere. Here all of the off-diagonals are in the range −0.1 to 0.6 which is surprisingly good considering that (i) no mechanism at all has been used to keep them apart and (ii) they are all sampling from a limited region of the input as shown in figure 19*b*. A few different views of this result are given in figure 20 where the plots in figure 20*a*,*c* are two- and three-dimensional views, respectively, of all the individual branch correlations and the plot in figure 20*b* shows a histogram of the same data to make the distribution of correlations more apparent.

It is worth mentioning that these results are gathered from branches that were allowed to continue replacing synapses for 1000 iterations. It is highly likely that terminating earlier (e.g. closer to the maximum of |Cov| as shown for one branch after 79 iterations in figure 18*a*) would produce smaller correlations between the branches. This is also a fruitful direction for future study.

## 6. Future work

Vision sensors have been built with embedded per-pixel processing units [59], which allow parallel, real-time feature computation in the sensor itself. Embedding retina-like computation in the sensor would further reduce redundancy whilst retaining information and producing custom representations (for motion, orientation, saliency, luminance, etc.).

Mammalian retinas send multiple representations of the environment down the visual pathway, how these are combined and affect learning of abstract versions of objects is still an open question. For example, motion signals could provide a hint to cortical regions so they have a less difficult job predicting how the environment will appear.

Different signals may affect learning in different ways, ‘slow’ neurotransmitters could provide windows for neurons to spike given sufficient ‘fast’ input (see §2.2). This interaction directly affects learning algorithms, such as STDP, since the ‘slow’ transmitter essentially supervises the time at which a post-synaptic neuron should spike. In this way, higher regions of cortex could drive learning of lower regions' outputs; or neurons in the same abstraction level, which react to the same input, could form bindings even if their receptive fields are far from each other.

The network architecture presented in §4 can be expanded with the addition of a winner-takes-all network connected to the output of the target layer. This would allow online classification with connectivity pre-trained using supervised learning.

We are also currently working on the addition of supervised learning mechanisms to the sparse-coded dendritic branches described in §5. We know that bAPs are generated in those parts of the dendritic tree closest to the soma when the associated neuron fires, and it has already been shown that these bAP signals can act as a modification or gating mechanism for synaptic plasticity [60,61]. Trying to stay close to these observations, a mechanism for gating the sampling process described in §5 would allow the dendritic branches to converge on features with good signal to noise on only a subset of the inputs received—perhaps, those associated with a training signal. This could, therefore, act as a fully local supervised learning mechanism and initial results on the same MNIST data are encouraging. We are still investigating the options for converting neuroscience observations into feasible computational mechanisms and hope to report in more detail soon.

## 7. Conclusion

Event-based vision offers a number of prospective advantages over conventional frame-based computer vision due to its inherent ability to focus limited computing resources on salient areas of the scene. Processing the spatio-temporal patterns of events that emerge in event-based vision is at an early development stage, but biology offers ample evidence that such systems can work well in practice.

Neuromorphic platforms are well-suited to large-scale event based processing, and SpiNNaker offers the flexibility of software to implement a range of neural (and non-neural) event processing models. These models may closely mimic biological processing, or be much more abstract in their biological inspiration. Event-based cameras are a good match to SpiNNaker's real-time spike processing capabilities.

Information theoretic approaches such as Maximum Entropy sampling can be emulated in event processing systems, and techniques such as synaptic rewiring open the possibility of achieving online unsupervised learning in near-optimal ways, a result that it is difficult to deliver using frame-based approaches due to the very high computational cost of training such networks.

## Data accessibility

The data and code used to generate the results presented in this paper are available from doi:10.17632/84pvnm3rj3.1 [62].
